# Assessing personality profile: A qualitative study from drug abuse rehabilitation policies

**DOI:** 10.1016/j.mex.2026.103831

**Published:** 2026-02-15

**Authors:** Eny Purwandari, Muhammad Japar, Setia Asyanti, Ro'iz Santria Giri, Ismiyati Yuliatun, Ema Madyaningrum, Nurlaela Widyarini

**Affiliations:** a Faculty of Psychology, Universitas Muhammadiyah Surakarta, Indonesia; b Balai Pemasyarakatan Class I Surakarta, Indonesia; c Psychology Clinic, Dr. Arif Zainudin Regional Mental Hospital, Surakarta, Indonesia; d Department of Mental Health and Community Nursing, Faculty of Medicine, Public Health and Nursing, Universitas Gadjah Mada, Indonesia; e Faculty of Psychology, Universitas Muhammadiyah Jember, Indonesia

**Keywords:** Personality, Substance abuse, Intervention, Public policy, Mental health

## Abstract

This article outlines a qualitative methodological framework for evaluating personality profiles within drug abuse rehabilitation systems and policy environments. The protocol combines expert-led Focus Group Discussions (FGDs) with a systematic analysis of social research inquiry (Litmas) documents to assess psychosocial risk and protective factors associated with recovery processes. Eight experts from psychiatry, psychology, correctional facilities, and national substance abuse rehabilitation policy participated in the focus group discussions. Thematic analysis was carried out to guarantee analytical transparency and cross-source triangulation. The protocol showcases how psychosocial characteristics can be systematically connected to rehabilitation practices and policy execution across health and correctional systems. This approach aims to assist in assessing the quality of rehabilitation programs and inform policy decisions based on evidence, especially in settings involving multiple sectors.

**Key methodological features of the protocol include:**

1. Integration of expert-based FGDs and institutional document analysis to support data source triangulation.
2. Analyzing rehabilitation practices and policy contexts in relation to personality-related constructs using systematic thematic methods.
3. A framework that can be reused to support the evaluation of rehabilitation systems in various sectors from a policy perspective.

## Graphical abstract

**Figure:**
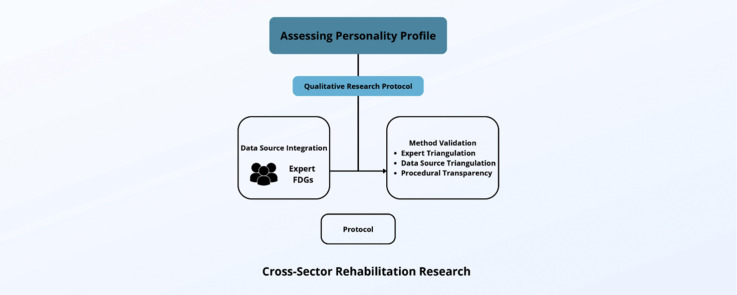

**Specifications table**

**Table:** 

Subject area	Psychology
More specific subject area	Personality profile of drug abuse
Name of your method	Assessing Personality Profile: A Qualitative Study from Drug Abuse Rehabilitation Policies
Name and reference of original method	Semi-structured interview guide, FGD guide, document analysis, Atlas.ti
Resource availability	None

## Background

Drug abuse constitutes a persistent public health and policy challenge, positioned at the intersection of mental health care, criminal justice, and social welfare systems. International diagnostic frameworks, including the Diagnostic and Statistical Manual of Mental Disorders 5 (DSM-5-TR), classify substance use disorders as mental health conditions requiring comprehensive and sustained intervention rather than solely punitive responses [1], as well as Indonesia’s national clinical guidelines such as the Guidelines for Classification and Diagnosis of Mental Disorders (Pedoman Penggolongan dan Diagnosis Gangguan Jiwa/PPDGJ) [2]. Accordingly, national drug policies emphasize integrated strategies combining prevention, law enforcement, and rehabilitation to address both individual vulnerability and broader societal impact.

National and regional studies indicate that drug abuse prevalence remains a persistent concern among adolescents and individuals of productive age, despite ongoing prevention efforts. Evidence from Southeast Asia and Indonesia shows sustained levels of substance use among adolescents and young adults, including university students, with psychosocial vulnerability and limited social support contributing to continued risk [[3], [4], [5]]. From a policy and criminal justice perspective, analyses of Indonesia’s drug control system highlight the substantial burden placed on law enforcement and correctional institutions, reinforcing the importance of rehabilitation-oriented approaches for reducing recurrence, supporting recovery, and promoting long-term public safety [6].

In Indonesia, rehabilitation policy is closely intertwined with legal and developmental considerations, especially in cases involving children and adolescents. Jurisprudence-based analyses emphasize that narcotics abuse among minors should be addressed through rehabilitation-oriented and restorative approaches rather than purely punitive measures [7,8]. This legal orientation reinforces the importance of rehabilitation systems that are responsive to psychological vulnerability, developmental stages, and social contexts, aligning justice objectives with mental health and social welfare principles.

Rehabilitation policies are implemented through multiple institutional arrangements, including health-based services coordinated by the National Narcotics Agency (BNN) and correctional-based rehabilitation delivered within prisons (Lapas) and community supervision centers (Bapas). Although these institutions operate under a shared national policy framework, they apply distinct operational logics and performance orientations [[8], [9], [10]]. Health-oriented rehabilitation prioritizes mental health treatment, counseling, and family involvement, whereas correctional-based rehabilitation emphasizes discipline, behavioral regulation, and social order. For policy evaluation, this institutional differentiation poses challenges in assessing coherence, coordination, and continuity of care across rehabilitation pathways.

Empirical psychological research in Indonesia consistently demonstrates that substance abuse and rehabilitation outcomes are influenced by personality-related factors, including patterns of delinquency, social control mechanisms, and value orientation. Studies show that weakened social control, maladaptive decision-making, and impaired self-regulation increase vulnerability to drug use and complicate recovery processes [[11], [12], [13]]. Relapse risk is further shaped by economic pressures, socio-cultural environments, stigma, and reintegration challenges following rehabilitation [14]. Furthermore, emotional distress and social isolation related issues like depression, anxiety, and stress are risk factors for behavioral changes, with a high prevalence among women [15]

Despite this growing evidence, policy-oriented research in Indonesia remains limited by the absence of standardized methodological protocols that systematically integrate psychological assessment with institutional and policy analysis. Many studies focus on program outcomes without clearly articulating how psychological factors are identified and linked to rehabilitation implementation processes [[16], [17], [18]]. This article addresses this methodological gap by presenting a qualitative protocol that integrates expert-based Focus Group Discussions with social research inquiry (Litmas*)* document analysis. By offering a structured and transparent methodological approach, the protocol supports qualitative evaluation of rehabilitation policies and facilitates evidence-informed decision-making across health and correctional sectors [19].

## Method details

### Study design

This study employed a qualitative case study protocol to document a systematic methodological approach for examining personality-related risk and protective factors within drug abuse rehabilitation systems. The qualitative case study design was selected to enable in-depth exploration of complex psychological and institutional processes embedded within specific rehabilitation and policy contexts, rather than to evaluate program effectiveness.

The case was defined at the system and institutional level, encompassing health-based rehabilitation services, correctional institutions, and community supervision settings operating under a shared national policy framework. This definition allows comparative analysis across institutional roles while maintaining a coherent unit of analysis.

The protocol integrates psychological perspectives, focusing on personality dynamics relevant to substance use and recovery, with institutional and policy-oriented analysis examining rehabilitation implementation across sectors. Emphasis is placed on procedural transparency and analytical traceability across all stages, including case definition, data collection, thematic analysis, and validation.

### Participants and research setting

The protocol was implemented with the participation of eight key informants selected to represent diverse institutional roles within Indonesia’s drug abuse rehabilitation system. Rather than focusing on service users, the protocol intentionally engaged expert informants who possess direct professional experience in designing, implementing, supervising, or evaluating rehabilitation programs. This approach aligns with the methodological objective of examining rehabilitation practices and policies through an institutional and policy-oriented lens.

The informants comprised a psychiatrist and a senior psychologist with extensive experience in clinical assessment and mental health treatment of individuals with substance use disorders, providing expertise on psychological vulnerability, personality dynamics, and recovery processes. Representatives from the National Narcotics Agency (BNN) contributed perspectives on national rehabilitation policy, program coordination, and regulatory implementation. Correctional Institution (Lapas) offered insights into correctional-based rehabilitation, including discipline-oriented programming, behavioral regulation, and institutional constraints. Correctional officers from community supervision centers (Bapas) contributed perspectives on post-release supervision, reintegration challenges, and continuity of care.

The research settings were located in Yogyakarta and Surakarta, two regions with established rehabilitation infrastructures and active collaboration between health and correctional institutions. These locations were purposively selected to reflect variation in institutional contexts, including hospital-based rehabilitation services, prison-based programs, and community supervision systems. This geographical and institutional diversity allowed the protocol to capture differences in rehabilitation orientation, implementation practices, and inter-agency coordination.

Participant selection followed a purposive expert sampling strategy based on predefined inclusion criteria: (1) formal professional roles within rehabilitation or correctional institutions; (2) direct involvement in drug abuse rehabilitation or policy implementation; and (3) a minimum of five years of relevant professional experience. This strategy ensured that the data generated were grounded in practical institutional knowledge rather than solely theoretical perspectives.

By integrating informants across multiple institutional levels and settings, the protocol facilitates expert triangulation and enhances methodological credibility. The composition of participants enables comparative analysis across health-based, correctional-based, and community-based rehabilitation contexts, supporting the protocol’s applicability for qualitative research and policy evaluation in multi-sector rehabilitation systems

### Sampling and recruitment

A purposive expert sampling strategy was employed to recruit informants with demonstrated professional expertise, formal decision-making roles, and direct involvement in drug abuse rehabilitation or policy implementation. Eligibility criteria included institutional affiliation, responsibility for rehabilitation planning or supervision, and substantial professional experience in relevant settings. This approach ensured the inclusion of multidisciplinary perspectives across health, correctional, and policy sectors, thereby enhancing the methodological relevance and applicability of the protocol for qualitative research on rehabilitation systems. This sampling strategy contributes to method validation by enabling expert triangulation across institutional roles, thereby supporting the credibility and applicability of the protocol in multi-sector rehabilitation and policy contexts.

## Research instruments

### Operational definition of personality profile

In this investigation, the concept of personality profile does not relate to stable trait-based personality constructs (e.g., the Big Five) or to established clinical personality disorder frameworks. It is operationally defined as a set of functional psychosocial characteristics that are relevant to substance abuse vulnerability, rehabilitation engagement, and recovery sustainability. The personality profile particularly includes psychological patterns such as emotional regulation, self-awareness, a commitment to alter, coping ability, anxiety and depressive inclinations, and perceived social support. These characteristics are context-dependent and adaptable, influenced by institutional settings, social connections, and past rehabilitation experiences.

This conceptualization emphasizes dynamic psychosocial processes over fixed personality traits, aligning with recovery-oriented perspectives in substance use research. Instead, the personality profile is used as an analytical tool to explore how psychological functioning influences rehabilitation practices, institutional logics, and policy implementation, rather than for diagnostic or psychometric purposes.

The research protocol utilised semi-structured interview guidelines and Focus Group Discussion (FGD) prompts as its primary qualitative tools. These instruments were designed to systematically obtain expert viewpoints on personality-related risks and protective factors within drug abuse rehabilitation systems, and their implications for institutional practice and policy implementation.

The development of instruments was guided by a thorough examination of academic research on substance use disorders, relapse prevention techniques, and rehabilitation methodologies. Specifically, psychological factors known to affect vulnerability and recovery were emphasized, such as anxiety, depression, resilience, self-awareness, commitment, emotional regulation, and perceived social support. Additionally, policy-focused research on rehabilitation administration, inter-agency collaboration, and care continuity was included to guarantee that the instruments accounted for institutional and regulatory aspects pertinent to policy assessment.

The semi-structured interview guidelines were organized into several thematic domains to guide data collection while allowing flexibility for in-depth exploration. These domains included:

(1) Personality-related characteristics, focusing on psychological patterns commonly observed among individuals undergoing rehabilitation; (2) Psychological risk and protective factors, exploring factors perceived to influence engagement, relapse, and recovery sustainability; (3) Rehabilitation practices and institutional approaches, examining how personality-related considerations are addressed within health-based and correctional-based rehabilitation settings; (4) Policy implementation and coordination, addressing perceived alignment, gaps, or overlaps between institutional mandates and rehabilitation policies; and (5) Continuity of care and reintegration, focusing on challenges and facilitators in post-treatment supervision, aftercare, and social reintegration.

FGD prompts were designed to encourage interactive discussion among multidisciplinary experts and to facilitate comparison of perspectives across institutional contexts. The prompts emphasized collective reflection on the integration of psychological assessment into rehabilitation practices, the feasibility of personality-informed policy approaches, and institutional challenges affecting program coordination and continuity of care. This format enabled participants to build on each other’s insights, supporting analytical depth and triangulation.

To ensure cultural relevance, clarity, and policy applicability, the instruments underwent an iterative refinement process. Draft versions were reviewed by senior researchers with expertise in clinical psychology, rehabilitation, and public policy. Revisions focused on contextualizing psychological constructs within Indonesian rehabilitation settings, simplifying terminology for accessibility to non-clinical stakeholders, and aligning question domains with policy evaluation needs. Minor adjustments were also made following pilot discussions to improve clarity, flow, and interpretability.

Overall, the use of semi-structured, thematically organized, and iteratively refined instruments enhances methodological rigor while maintaining flexibility. The instruments are designed to be adaptable and reusable for qualitative studies examining drug rehabilitation systems and policy implementation across diverse institutional and cultural contexts.

The thematic domains embedded in the interview guidelines and FGD prompts directly informed the analytical structure presented in Table 1, Table 2, Table 3. Specifically, domains related to professional roles and institutional contexts underpin the participant characteristics summarized in Table 1, while domains addressing personality-related risk and protective factors guided the development of codes and themes presented in Table 2. Policy implementation and continuity-of-care domains further supported cross-source triangulation between FGD data and Litmas document analysis, as reflected in Table 3.

**Table 1:** Characteristics of FGD Participants.

Subject	Professional Role	Age (years)	Years of Experience	Educational Background
FM	Psychiatrist	40	12	Medical Specialist in Psychiatry
K	Senior Psychologist	69	43	PhD in Psychology
SH	Head of Correctional Center (Bapas)	54	29	Master in Management
SuH	Head of Adult Client Guidance Section (Bapas)	39	19	Bachelor of Law
PS	Head of Narcotics Prison (Lapas)	55	30	Master of Law
D	Prison Staff (Lapas)	32	12	Bachelor of Law
FKDM	National Narcotics Agency (BNN)	41	17	Master of Hospital Administration
RAS	National Narcotics Agency (BNN)	36	13	Master of Psychology

**Table 2:** Main Themes Derived from FGD Analysis.

Theme	Categories	Key Codes
Rehabilitation	Approach & strategy	Holistic approach; counseling; follow-up; community care
	Support & development	Self-improvement; reintegration; communication
	Challenges	Data privacy; confusion; sustainability
Policy	Policy response	Monitoring; effectiveness; critique
Drug-related issues	Psychological aspects	Mental health; self-awareness; commitment
Addiction	Socio-economic impact	Financial stress; avoidance
Life of people with addiction	Social challenges	Stigma; loneliness; trauma; social interaction

**Table 3:** Themes from Social Research Inquiry (Litmas) Documents.

Theme	Categories	Codes
Risk factors	Social environment	Peer drug-use networks
	Family	Lack of parental attention; broken home
	Economic	Financial pressure; heavy workload
	Psychological	Anxiety; depression; restlessness
Substance use pattern	Type of substance	Methamphetamine; cannabis; sedatives; amphetamine
	Usage pattern	Experimental; regular; dependence
Impact of drug abuse	Individual	Mental disorders; aggressive behavior
	Social	Criminal behavior; stigma
Rehabilitation efforts	Prison-based rehabilitation	Personality and independence development
	BNN rehabilitation	Counseling; psychoeducation; family support
	Aftercare (Bapas)	Client supervision; mentoring

This research employs standardized operational definitions for key institutional terms in order to maintain conceptual clarity and methodological consistency throughout the study. These terms derive from Indonesia's correctional, social rehabilitation, and narcotics control systems and may not have direct counterparts in other national or disciplinary frameworks. As a result, each term is defined within its established legal and functional context to prevent misinterpretation, ensure transparency in the research process, and improve the reproducibility of the method in comparable institutional environments.

1. Litmas (Correctional Social Inquiry). A structured social research report conducted by Community Guidance Officers at Community Supervision Centers (Balai Pemasyarakatan/Bapas) evaluates the social, psychological, and environmental conditions of correctional clients, informing guidance, rehabilitation, and decision-making within the correctional system. A structured social research report conducted by Community Guidance Officers (PK) at Community Supervision Centers (Balai Pemasyarakatan/Bapas) to assess the social, psychological, and environmental conditions of correctional clients, serving as a basis for guidance, rehabilitation, and decision-making within the correctional system.
2. PK (Community Guidance Officers), also known as PK officials, are functional professionals within the Correctional Institution (Bapas) whose primary responsibilities include offering assistance, providing guidance, overseeing supervision, and conducting community research for correctional clients. Prisoners' Kinsfolk play a strategic role in the Indonesian correctional system as they act as intermediaries between clients, families, communities, and the legal system. PKs play a strategic role in the Indonesian correctional system as they act as liaisons between clients, families, communities, and the legal system.
3. Bapas (Community Supervision Center), is a correctional institution responsible for providing supervision, guidance, and social rehabilitation services to correctional clients during pre-adjudication, post-adjudication, and reintegration phases.
4. BNN (National Narcotics Agency), is a national government agency responsible for the prevention, control, and eradication of narcotics and other addictive substance abuse, including coordination of rehabilitation services for individuals affected by substance use disorders.
5. Lapas (Correctional Institution), is a secure correctional facility where prisoners and juvenile offenders undergo custodial rehabilitation as part of the criminal justice process.

## Data collection procedure

The protocol employed a two-source data collection strategy to enhance analytical depth and methodological rigor. Primary data were collected through a 150-minute offline Focus Group Discussion (FGD) involving all key informants. The FGD was facilitated by the principal investigator to ensure consistency with the research objectives and was supported by trained research assistants responsible for time management, documentation, and observational note-taking. The session followed a structured yet flexible guide aligned with predefined thematic domains, allowing participants to elaborate on issues relevant to their institutional roles.

All discussions were audio-recorded with prior informed consent**,** and detailed field notes were taken to capture contextual information, non-verbal interactions, and key points of consensus or divergence. These complementary data sources supported interpretive accuracy during analysis.

Secondary data consisted of social research inquiry (Litmas) documents obtained from participating institutions. These documents provide standardized assessments of individuals undergoing rehabilitation, including psychosocial backgrounds, behavioral histories, and institutional evaluations. The inclusion of litmas documents enabled triangulation between expert perspectives and institutional records, strengthening the credibility and transferability of the protocol across rehabilitation and policy contexts.

## Data processing and analysis steps

Data processing and analysis followed a systematic, step-by-step thematic analysis protocol designed to ensure transparency, analytical traceability, and methodological rigor. The procedure began with verbatim transcription of all audio-recorded Focus Group Discussions (FGDs). Transcriptions were checked against recordings for accuracy and completeness, and anonymized prior to analysis to protect participant confidentiality.

In the first analytical phase, initial open coding was conducted to identify discrete units of meaning within the data. Coding focused on psychological constructs, social contexts, and policy- or institution-related issues relevant to drug rehabilitation. Codes were generated inductively while remaining informed by the predefined thematic domains embedded in the research instruments.

Subsequently, codes were grouped into higher-order categories representing broader analytical domains, including personality dynamics, rehabilitation practices, and institutional or policy perspectives. This categorization process involved constant comparison across data segments to ensure internal consistency and conceptual clarity.

The third phase involved the development of overarching themes through iterative team discussions. Multiple researchers reviewed coded data and emerging categories, refining thematic definitions through consensus-building and peer debriefing. Discrepancies were resolved through discussion rather than individual adjudication, enhancing dependability and reducing interpretive bias.

To strengthen credibility, triangulation was conducted by comparing themes derived from FGD data with findings from social research inquiry (Litmas) document analysis. Convergent and divergent patterns were systematically examined to assess the robustness of the analytical structure.

Throughout the process, Atlas.ti software was used to support data organization, coding, memo writing, and audit trail development. Analytical rigor was ensured by adhering to established qualitative trustworthiness criteria, including credibility, transferability, dependability, and confirmability.

## Data saturation

Thematic saturation was achieved during the analysis process. No substantively new codes or themes emerged after iterative coding and comparison across FGD transcripts and Litmas documents. Recurrent patterns related to psychological risk factors, recovery-supportive characteristics, and institutional rehabilitation approaches were consistently observed across data sources. This saturation indicates that the analytical categories were sufficiently developed to support the objectives of the protocol and reinforces the adequacy of the sample size for a qualitative methods article.

## Methodological inovation and contribution

This study's methodological innovation lies in the systematic integration of psychological, institutional, and policy-oriented data sources, using established qualitative methods such as Focus Group Discussions (FGDs), document analysis, and thematic analysis within a single analytical framework.

The protocol initially facilitates a structured integration of psychological personality constructs with institutional and policy documents. This approach connects psychosocial characteristics with rehabilitation practices and policy across both health and correctional systems, rather than looking at psychological factors individually. This integration enables qualitative psychological insights to directly influence policy analysis and institutional assessments.

The protocol instead utilizes social research inquiry (litmas) documents as psychosocial analytical data, rather than viewing them solely as administrative or procedural records. The protocol transforms these documents into a valuable secondary qualitative data source by systematically coding literature content for psychological risk and protective factors.

The design of cross-sector expert FGDs functions primarily as a method validation tool, rather than just a data collection approach. The inclusion of experts from psychiatry, psychology, correctional institutions (Lapas and Bapas), and national policy agencies (BNN) facilitates real-time triangulation of psychological, institutional, and policy viewpoints. This configuration enables a critical evaluation of the consistency, relevance, and applicability of analytical categories across sectors, thereby enhancing methodological credibility and transferability.

The proposed protocol's methodological novelty is brought about by these features collectively. This method's value lies in its reusability for the qualitative assessment of rehabilitation systems, especially in policy environments where psychological assessments, institutional practices, and regulatory frameworks need to be analysed simultaneously.

## Method validation

Protocol validation was conducted through expert triangulation in FGDs involving multidisciplinary stakeholders. Cross-institutional representation enhanced the credibility and applicability of the protocol. Participant characteristics are summarized in Table 1.

Table 1 demonstrates expert heterogeneity as a core validation strategy, ensuring that the protocol is examined across clinical, correctional, and policy-oriented perspectives. Table 1 supports method validation by demonstrating the multidisciplinary composition of expert informants involved in the Focus Group Discussions. The inclusion of professionals from psychiatry, psychology, correctional institutions (Lapas and Bapas), and the National Narcotics Agency (BNN) ensures that the protocol is informed by diverse institutional perspectives. This heterogeneity strengthens expert triangulation, validating that the protocol is applicable across different rehabilitation and policy contexts rather than being limited to a single disciplinary viewpoint. Based on the FGD attended by various experts, the themes summarized in Table 2 were obtained.

Table 2 contributes to method validation by illustrating the systematic output of the thematic analysis process derived from the FGDs. The structured presentation of themes, categories, and codes demonstrates that the protocol is capable of consistently organizing complex qualitative data into coherent analytical domains. This table validates the analytical stability and internal consistency of the method, indicating that the protocol produces reproducible thematic structures when applied to expert-based qualitative data. FGD data was validated by data from social research inquiry (Litmas). Table 2 confirms analytical consistency by showing that complex qualitative data can be systematically organized into stable themes, categories, and codes across expert discussions.

Table 3 further strengthens method validation by providing evidence of data source triangulation through the analysis of social research inquiry (Litmas) documents. The alignment between themes identified in Litmas documents and those emerging from FGDs confirms that the protocol captures personality-related risk and protective factors across independent data sources. This convergence validates the transferability and robustness of the method when applied to institutional documentation beyond interview-based data. Table 3 provides cross-source validation, demonstrating convergence between expert-derived themes and independent institutional documentation, thereby supporting the robustness and transferability of the protocol.

Table 1, Table 2, Table 3 demonstrate that the proposed protocol is validated through expert diversity, analytical rigor, and cross-source convergence, supporting its use as a reliable and reusable qualitative method for research on drug abuse rehabilitation and policy integration.

Method validation was achieved through a combination of expert triangulation, data source triangulation, and procedural transparency. Validation focused on ensuring that the protocol reliably captures personality-related constructs within rehabilitation systems across institutional contexts.

Expert triangulation was established through the inclusion of informants from diverse professional and institutional backgrounds, including clinical, correctional, and policy-oriented roles (Table 1). This diversity enabled cross-validation of perspectives and strengthened the credibility of the analytical domains. Data source triangulation was conducted by systematically comparing themes derived from FGDs with findings from social research inquiry (Litmas) documents (Table 2, Table 3), allowing convergence and divergence across sources to be explicitly examined.

Procedural validation was further supported by standardized data collection procedures, verbatim transcription, team-based coding, and consensus-driven theme development. The use of Atlas.ti facilitated transparent data management and the creation of an audit trail. Together, these strategies ensure that the protocol demonstrates credibility, dependability, and applicability, supporting its reuse in qualitative research on rehabilitation systems and policy-oriented mental health studies.

## Limitations

Several methodological limitations of this protocol need to be taken into account when evaluating its suitability for use. Initially, participation was restricted to only eight experienced consultants, representing The Ministry of Health, The Minsitry of Immigration and Correction, Expert in Psychology and Medicine. The perspectives captured may not fully represent all institutional variations across regions or rehabilitation settings in Indonesia, such as Social Rehabilitation Center for Drug Abuse (BRSKPN) and rehabilitation institution non-government.

The analysis was also dependent on the accuracy and thoroughness of social research inquiry (litmas) documents, whose quality may differ between institutions due to varying documentation methods. The variability in question may affect the extent of psychosocial analysis that can be undertaken using secondary institutional data.

The protocol primarily concentrates on the viewpoints of experts and institutions, rather than firsthand accounts from individuals receiving rehabilitation. Future applications of the protocol could incorporate service-user perspectives to increase analytical depth and triangulation, although the current design is suitable for policy and method development.

This study is designed as a qualitative research protocol, which does not seek to achieve statistical generalizability. The significance of the contribution stems from its provision of analytical transparency, procedural rigour, and reusability in the qualitative assessment of rehabilitation systems, rather than in quantifying causal associations.

## Related research article

None

## For a published article

None

## Ethics statements

This study was conducted in accordance with the ethical standards of research involving human participants. Ethical approval was obtained from the Health Research Ethics Committee of the Faculty of Medicine, Universitas Muhammadiyah Surakarta (Approval No. 5366/B.2/KEPK-FKUMS/X/2024). Permission to conduct the study was also granted by the relevant governmental and institutional authorities.

All participants were informed about the purpose, procedures, and voluntary nature of the study prior to data collection. Written informed consent was obtained from all participants. Confidentiality and anonymity were ensured throughout the research process, and all data were used solely for academic purposes. The study complied with the ethical principles outlined in the Declaration of Helsinki and the American Psychological Association’s Ethical Principles of Psychologists and Code of Conduct.

## CRediT author statement

**Eny Purwandari**: Conceptualization, writing original draft, funding acquisition, supervision, project administration, data curation; **Muhammad Japar**: Validation, Writing–review & editing; **Setia Asyanti**: visualization, writing–review & editing; **Ro'iz Santria Giri**: resources; **Ismiyati Yuliatun**: Resources. **Ema Madyaningrum**: Conceptualization, data curation, resources; **Nurlaela Widyarini**: Conceptualization, data curation, resources.

## Declaration of generative AI and AI-assisted technologies in the manuscript preparation process

During the preparation of this work, the author(s) used ChatGBT in order to improve the readability, language, and structural flow of the manuscript. After using this tool/service, the author(s) reviewed and edited the content as needed and take(s) full responsibility for the content of the publication.

## Declaration of competing interest

The authors declare that they have no known competing financial interests or personal relationships that could have appeared to influence the work reported in this paper.

## Data Availability

The data that has been used is confidential.
